# Supplementary material for: Mildly Pasteurized Whey Protein Promotes Gut Tolerance in Immature Piglets Compared with Extensively Heated Whey Protein
Source: Nutrients. 2020 Nov 4;12(11):3391. doi: 10.3390/nu12113391 (PMC7694243; doi:10.3390/nu12113391)
Supplement: Supplementary file 1 [file nutrients-12-03391-s001.zip › nutrients-973148-supplementary.pdf]

Table S1: Enteral & Parenteral Nutrition. Piglets received minimal enteral nutrition for 5 days based in increasing dose, with additional parenteral nutrition support. Additional enteral boluses are also indicated.

	Enteral Nutrition	Parenteral Nutrition	Extra bolus
**Day 1:**	6 ml/kg/3h	4 ml/kg/h	-
**Day 2:**	8 ml/kg/3h	4 ml/kg/h	-
**Day 3:**	8 ml/kg/3h	4 ml/kg/h	5% galactose : 15 ml/kg (9 am)
**Day 4:**	10 ml/kg/3h	4 ml/kg/h	10% lactose : 15 ml/kg (9 am)
			X-ray contrast fluid : 4 ml/kg (6 pm)
**Day 5:**	10 ml/kg/3h	4 ml/kg/h	5% lactulose/mannitol : 15 ml/kg 3 hrs before euthanasia
			last bolus of EN : 15ml/kg 1 hr before euthanasia

Table S2: Fecal assessment. Fecal assessment was performed twice a day blinded for diet. Date and time for first meconium after birth were also registered. For analysis, score ≥3 was classified as a piglet with diarrhea.

Score	feces
0	No stool
1	Firm feces
2	Pasty feces
3	Droplets of watery feces / diarrhea
4	Moderate amounts of diarrhea
5	Large amounts of diarrhea

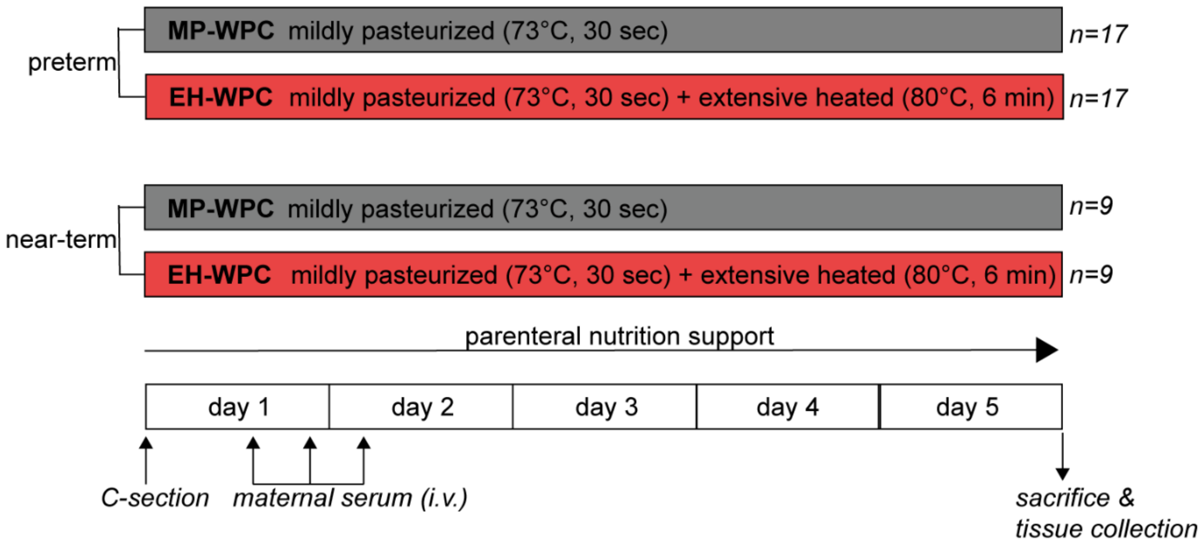

Figure S1: Experimental set-up. Preterm and near-term piglets were delivered by caesarean section at 90% gestation (n=34, 2 litters) and 96% gestation (n=18, 1 litter) respectively. Piglets from each litter were stratified according to birthweight into 2 groups of enteral diets, 1) MP-WPC and 2) EH-WPC. In addition to the enteral nutrition, all piglets received parenteral nutrition support from day 1 to day 5. For passive immunization, piglets received three doses of maternal serum (iv). On day 5 the piglets were euthanized, followed by collection of tissue / GI tract content samples.

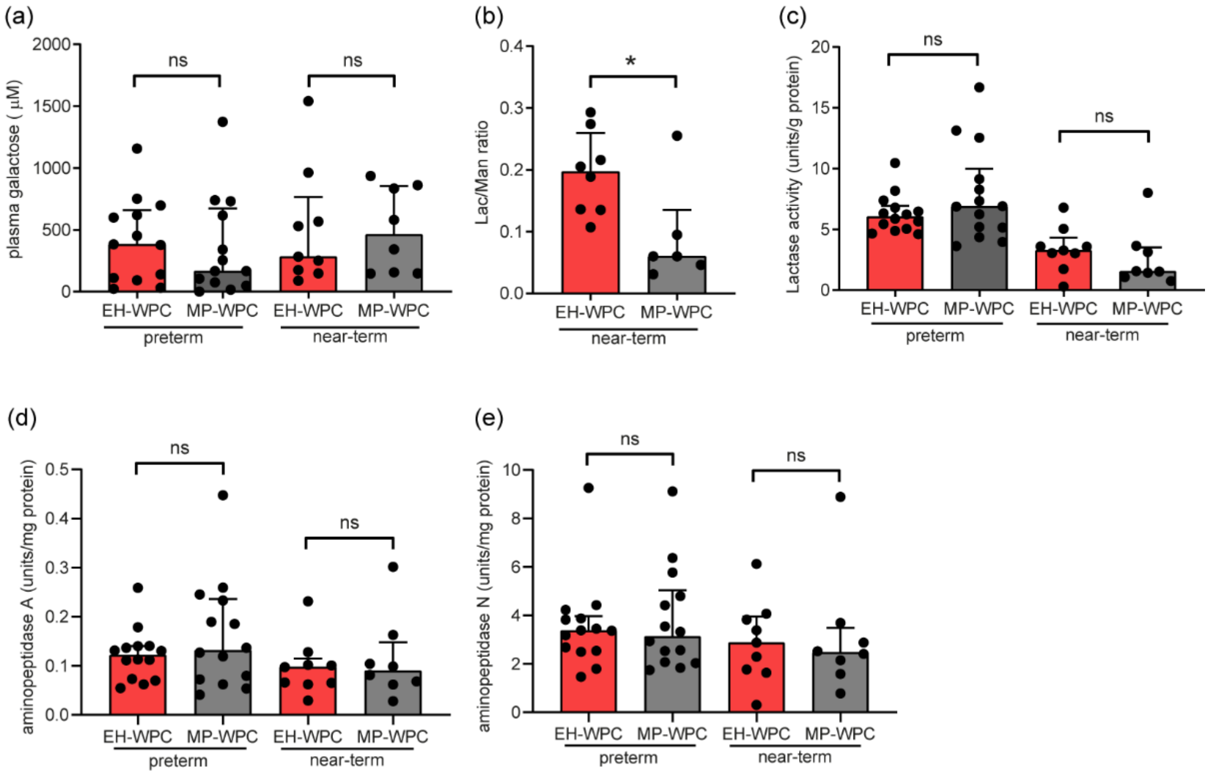

Figure S2: Absorption (**a**) Increase in plasma galactose (µM) 20 min after an oral bolus of galactose on day 3. (**b**) Urinary lactulose/mannitol ratio measured 3 h after an oral bolus of lac/man on day 5. (**c-e**) Brush border enzyme activity levels determined in mid small intestinal tissue with (**c**) lactase-phlorizin hydrolase, (**d**) aminopeptidase A and (**e**) aminopeptidase N activity. n=14 for preterm piglets, n=8-9 for near-term piglets.*p<0.05, ns= non-significant based on Mann-Whitney test between EH-WPC and MP-WPC.

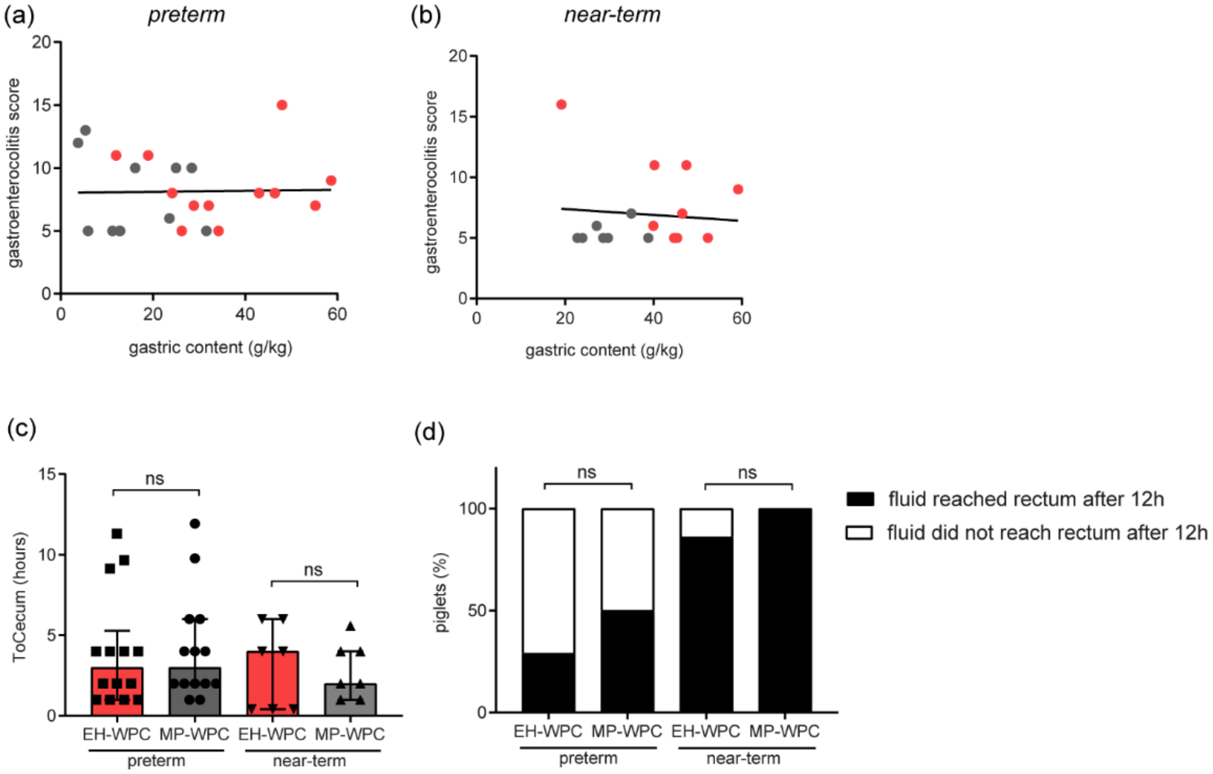

Figure S3: Gastric content, gastro-enterocolitis and colonic transit time. (**a-b**) Gastric content volumes (g/kg) with total gastro-enterocolitis score for (**a**) preterm and (**b**) near-term piglets. Correlation tested with Spearman's correlation test. (**c**) Time (h) of contrast fluid to first appear in the cecum, determined by x-ray analysis, with (**d**) % of piglets in which the contrast fluid appeared in the rectum after 12 h.

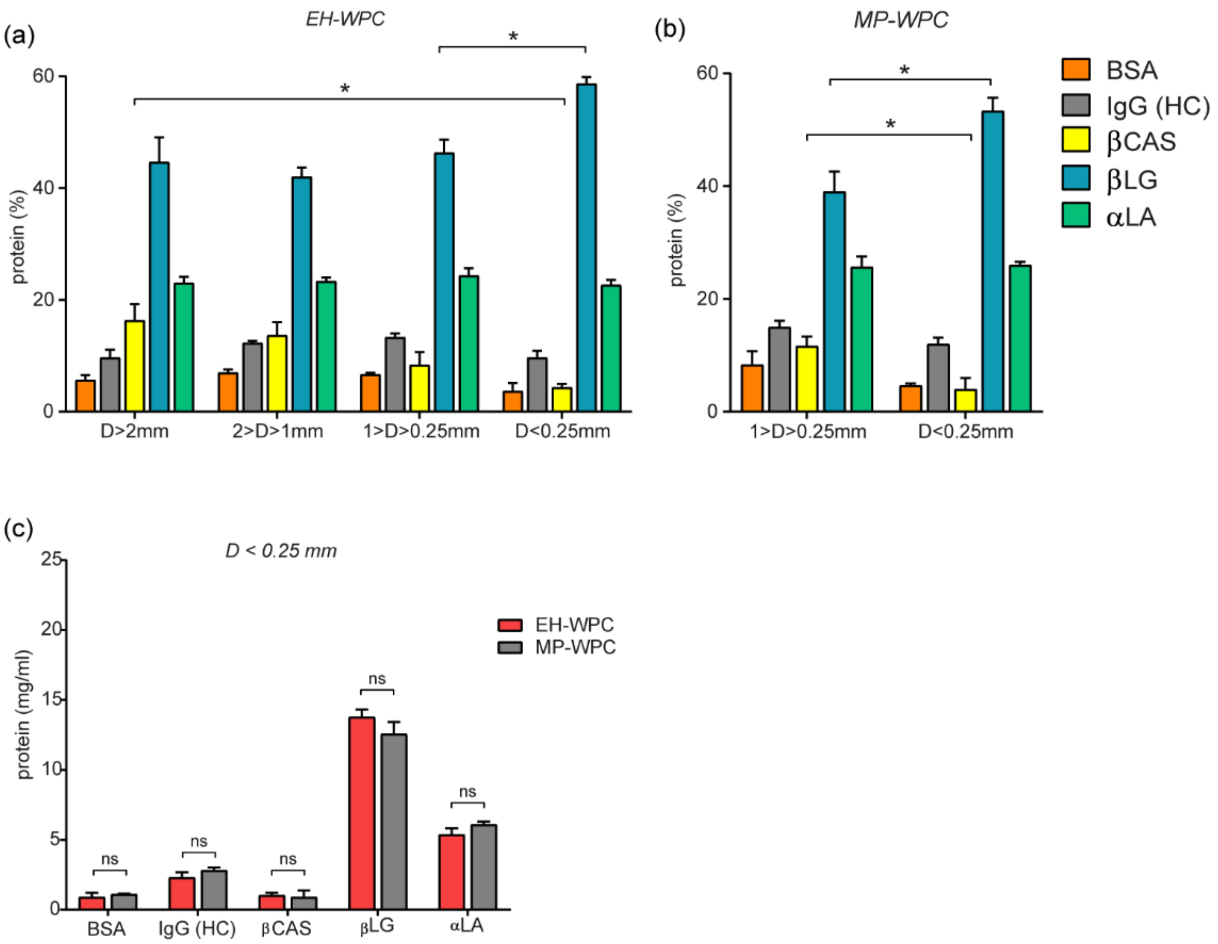

Figure S4: ***in vitro*** **protein analysis.** (**a**-**b**) Relative protein abundance per fraction (% of total protein in fraction) calculated based on SDS-PAGE separation for (**a**) EH-WPC and (**b**) MP-WPC. n=3, *p<0.05 based on one-way ANOVA with Tukey's post-test, comparing protein abundance in all fractions for each specific protein. (**c**) Protein concentrations calculated based on SDS-PAGE separation for the digested fraction with a particle size D < 0.25 mm. n=3, ns = non-significant based on t-test between EH-WPC and MP-WPC. BSA = bovine serum albumin, IgG (HC) = immunoglobulin G (heavy chain), βCAS = β-casein, βLG = β-lactoglobulin, αLA = α-lactalbumin.
